# The nature, sequence and duration of professional activities of Emergency Medical Service providers: An observational study to evaluate quality of care using the steps in the EMS care process as described by the SPART model

**DOI:** 10.1371/journal.pone.0311946

**Published:** 2024-12-02

**Authors:** Bert Dercksen, Michel M. R. F. Struys, Wolter Paans, Fokie Cnossen

**Affiliations:** 1 Department of Anesthesiology, University Medical Centre Groningen, University of Groningen, Groningen, The Netherlands; 2 UMCG Ambulance Care, Tynaarlo, The Netherlands; 3 University of Groningen-Faculty of Medical Sciences, Groningen, The Netherlands; 4 Faculty of Science and Engineering Artificial Intelligence — Bernoulli Institute, University of Groningen, Groningen, The Netherlands; 5 Hanze University of Applied Sciences-Nursing Diagnostics and Centre of Expertise Healthy Ageing, Groningen, The Netherlands; University of Hong Kong, HONG KONG

## Abstract

**Background:**

The quality of care given by EMS staff is in part monitored by routine review of deployment reports that the providers must complete after each deployment. The assessment consists of determining whether a deployment was performed in a medically correct manner and thus whether the clinical reasoning process was appropriate. The time used during the deployment is also measured, as there are often time limits for performing different parts of the deployment and these must be adhered to. One might question whether measuring the time spent really gives insight into the quality of care, and if it might not be more useful to see how that time was spent. We therefore conceived a study to map the EMS care process and evaluate this process in relation to the time used.

**Methods:**

We used a focused ethnographic study design. Video recordings of EMS deployments in combination with semi-structured interviews of the EMS providers involved, were thematically analysed. This led to insights into the EMS care process and the embedded clinical reasoning. By categorising the professional activities we identified we were able to define a model that describes in general the EMS care process. We reported the first part of this study in which we developed this so called SPART model in a separate paper. In this second part of the study we determined the execution time within a deployment and measured the frequency of occurrence and the duration of the identified activities. We interpreted the operational content and the time variables both qualitatively and quantitatively. We also determined the distribution of activities over the three deployment operational periods (response, on-scene and transport period).

**Results:**

Using the SPART model, we gained insight into the different activities’ nature, order, and duration. We could qualitatively judge the effectiveness of the clinical reasoning process, i.e., the quality of care delivered. Generally, the studied cases were followable, and the clinical reasoning process was medically logical. The diagnostic process sometimes continued after the clinical decision, which was not medically logical. Remarkably, this never changed the clinical decision. Although this could negatively affect the quality of care, we found no clinical evidence that this was the case.

**Conclusion:**

Our findings demonstrated that the quality of care in EMS can be measured by using the SPART model to evaluate EMS deployments. We concluded that qualitative judgment was more important than quantitative evaluation. Interpreting the order of different activities led to the clearest understanding of the clinical reasoning process. It was concluded that knowledge of the exact time used per activity and, in total, had the least impact on understanding the clinical reasoning process.

## Introduction

Routine retrospective evaluation of Emergency Medical Services (EMS) care by a supervising physician is an established part of monitoring the quality of care in many EMS systems. This is mainly done by inspecting (Electronic) Deployment Reports (EDR) [1–5]. One then assesses whether the professional activities carried out are traceable and medically coherent, whether the course of action followed is correct and whether the right conclusion has been reached, allowing insight into the quality of care given. Quality of care in EMS can be represented by both subjective and objective determinants. An example of a subjective evaluation of quality of care is the standard enquiry of patient satisfaction with the care provided. Examples of objective evaluation of the delivered care are the comparision of the EMS diagnosis and the hospital diagnosis or the time necessary to reach the patient, the time used on scene and the transportion time to a medical facility.

In many countries, national laws and regulations prescribe time limits for the response, on-scene and transport periods in EMS deployments, placing time constraints on life-threatening and non-life-threatening cases. So time spent is often used to measure the quality of care, conveniently ignoring how this time is used. Whether this always provides accurate insight into the quality of care is debatable. Sometimes, extra time may be justified to optimise the clinical reasoning process to arrive at a more precise clinical decision in a complex case. The clinical reasoning [6–8] process is a cognitive process in which the professional tries to answer the patient’s request for help by obtaining (medical) information through history-taking and examination, interpreting it and processing it into a clinical diagnosis. This diagnosis is transformed into a clinical decision in accordance with the patient’s wishes and the given options for implementing treatment. In other words, it is decided what the best response is to the patient’s request for help. Going through this process takes time. Minimising the emphasis on time as a quality factor could reduce the risks of cognitive biases [9]. The potential friction between the speed of action and comprehensiveness is one of the characteristics of Emergency Medical Services’ field of activity [10]. Emergency Medical Services responders often have to start treatment of the clinical symptoms before a complete and definitive diagnosis has been made, whereas ideally, a complete diagnosis precedes a treatment plan. In acute cases, the optimal strategy is to treat disturbed vital parameters immediately, i.e., even before the diagnostic process is complete. In other less life-threatening cases, making a proper working diagnosis before starting the therapy is better. However, even if in these cases, the timespan for this is not unlimited. Several studies addressing the factor time in EMS relate patient outcomes to the length of the various deployment phases [11, 12]. However, other research demonstrates that time spent during a deployment seems less important [13–16].

To our knowledge, no literature exists addressing the content of the EMS care process (i.e. what do EMS providers do during a deployment?) in general, and in relation to the time it takes. Knowledge about the EMS care process provides insights into the clinical reasoning process executed by EMS providers. Therefore we conceived a study to research the EMS care process. We published the first part of this study in 2021 [17] in which we demonstrated that EMS providers tend to follow a standard workflow to arrive at a clinical decision. In a continuous process, they collect, interpret and process information about the specific medical problem, the patient’s general history, and his current overall medical status in a step-by-step manner. The emerging diagnostic considerations are weighted, collated and verified with additional questions and examination. Finally, a working diagnosis is reached, and a clinical decision is formulated, eventually leading to therapy and transfer. The SPART model was developed based on these findings. It describes the sequential phases of the EMS care process. In this paper we present the second part of the research in which we have tried to reveal the relationship between the factor time and the EMS workflow.

### EMS in the Netherlands

Emergency medical care in the Netherlands is provided by specialised Ambulance Nurses (AVP) or Bachelor-degree qualified paramedics (BMH) and specialised Ambulance Drivers (ACH). Each ambulance is operated by a crew of two: an AVP or BMH assisted by an ACH. EMS care is highly protocolised. EMS personnel across the country must adhere to more than 70 protocols. To ensure a high standard of care, EMS staff must undergo continuous medical education and training after their initial instruction. A team operates autonomously during a deployment, but a supervising doctor is available by phone 24 hours a day for consultation. Afterwards, the quality of care is assessed by the supervising physician through, among other things, evaluation of the (Electronic) Deployment Reports.

### The SPART model

The SPART model describes the structure of the EMS care process. The EMS care process is comparable to the nursing process [18] but has distinctive characteristics. The model divides the EMS process into ten consecutive imaginary steps. The sequence of these steps is flexible with steps occasionally skipped or repeated. Also, the order of execution is variable.

Nevertheless, at the end of almost every deployment, all steps will have been completed. Within each step, certain professional activities can be identified. The activities within a step constitute one category. The ten steps or categories are represented by the acronym SPART. Each letter represents one step or category.

#### Start

Each deployment is initiated by information collated in the Start category. This information is obtained during the phone call between the caller and the dispatch centre and passed to the ambulance personnel. Often, this information initiates a cognitive process in which the EMS providers generate an initial potential scenario.

#### Situation

When arriving on the scene, the ambulance crew will often get a first intuitive impression of the patient and their situation. This information is summarised within the Situation category.

#### Presentation or presenting complaint

Logically, the next step is to gather information about the patient’s primary complaint. Usually, this is typically done by asking specific questions about the nature of this complaint and conducting a focused examination. These actions characterise the Presentation or Presenting complaint category.

#### Prologue

In the Prologue step, information is collected about the “history” of the complaint: “How and when did it start?”“Is it a recurrent complaint?”

#### Anamnesis and assessment

To gather information about the patient’s general medical status, a general history is taken (Anamnesis), and a general medical examination is executed (Assessment).

#### Recapitulation and reasoning

A review will usually be executed at some point during the deployment. “Do we have enough information or is something missing?”, “What can be concluded?”, “Are we able to generate a working diagnosis and differential diagnosis?” will typically be asked during this step.

#### Resolution

Ultimately, the process leads toward the formation of a clinical decision.

#### Treatment

The clinical decision will generally lead to the initiation of therapy.

#### Transfer

The last step in any deployment is the transfer step.

S1 Table and this animation (https://youtu.be/BG-huO8yssw) provide a more detailed explanation of the model.

### Goal of this study

To better understand the nature and time spent on the clinical reasoning process in EMS, we further analysed the dataset we used to develop the SPART model [17] (the dataset is available as S1 Data). We identified professional activities utilising this model and qualitatively analysed this data, focusing on followability and medical appropriateness. We also quantitively determined the sequence and duration of activities.

We formulated two research questions to be answered.

1. Is it possible to use the SPART model to identify the sequence of the professional activities of EMS providers and judge if they are systematic and coherent, and does this provide insight into the clinical reasoning process?
2. Is measuring the duration and identifying the distribution over the deployment periods of professional activities helpful in judging the quality of provided EMS care?

## Methods

### Study design

We used a study design based on focused ethnography principles consisting of non-participant real-life video observations of EMS deployments combined with a content analysis by semi-structured interviews of the involved EMS providers. Focused ethnography involves collecting data focusing on a specific aspect of a community’s activity within a limited period of time [19, 20]. The non-participant observation method [21] implies no interaction between the researcher and the observed participant during the observation.

### Setting and data collection

The study was executed in the northern part of the Netherlands. Video data collection took place between October 8^th^ 2018 and December 23^th^ 2018. The participants (EMS providers who voluntarily participated in the study) wore video-recording glasses (Ricon 1080p, Ivue Camera, Sugar City ID, USA) [22] during their daily work. The providers filmed their assignments with these video glasses from the deployment assignment until the end (e.g., transfer to a hospital). During the deployments, a research assistant (final year nursing student) accompanied and assisted each participant in study-related tasks, such as operating the glasses. After arrival on the scene, the research assistant asked patients for informed consent to videotape the deployment and data use. Only videos for which informed consent was obtained from both the EMS provider and the patient were included in the analysis. Immediately after a deployment, participants were interviewed using the recordings as a memory cue to reveal their diagnostic activities, goals, intentions, working hypothesis and more. The interviews were digitally recorded and, in a later stage, verbatim transcribed. Each interview was stored together with the matching video recording for further processing.

We reported our research following the SRQR guideline [23].

**Fig 1:**
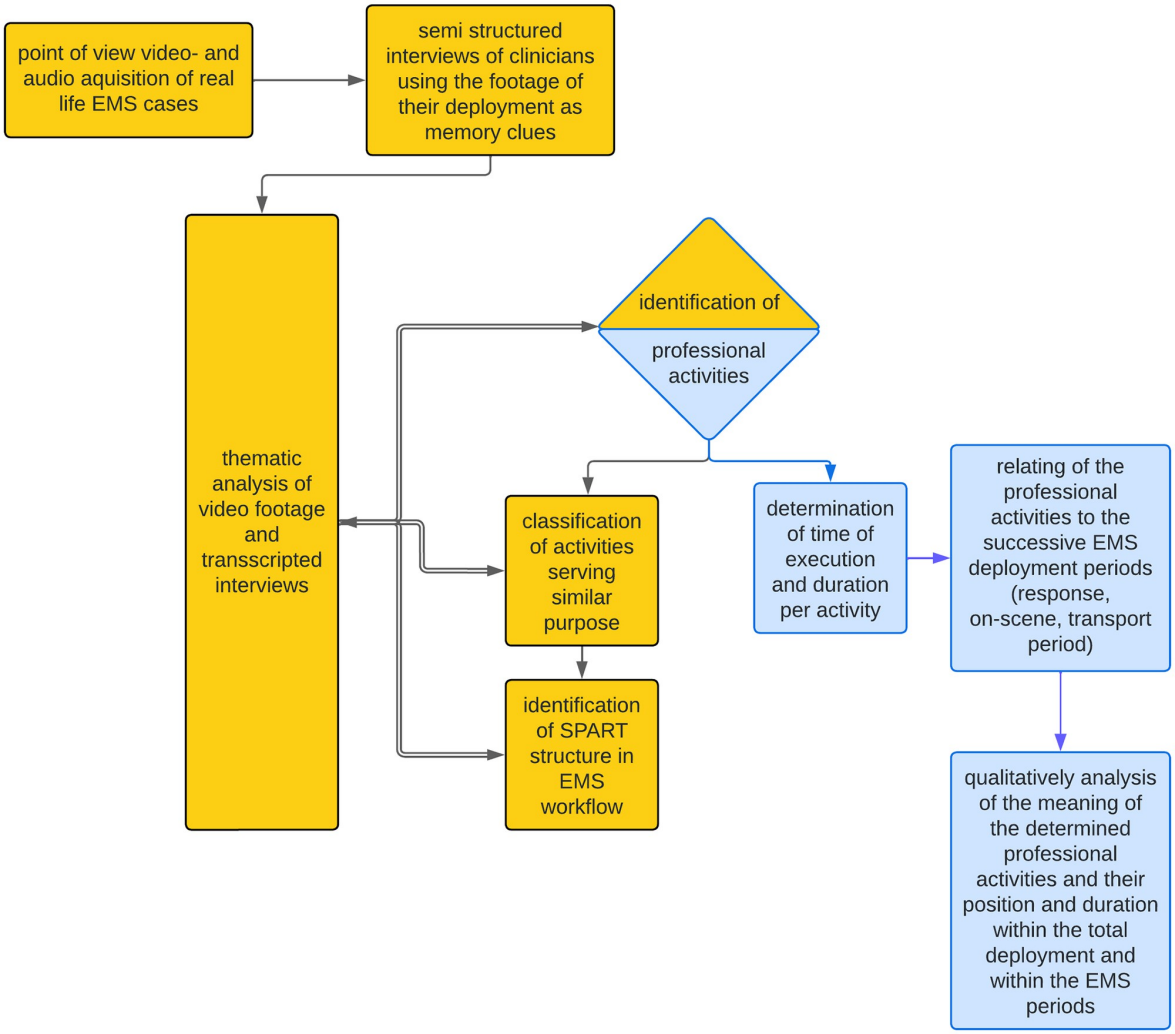
Research phases. Yellow is the SPART model development. Blue is the determination of the relation between time and EMS care process.

Fig 1 illustrates the research phases. Yellow represents the part of the research in which we defined the SPART model, blue represents the part of the study that aimed to clarify the relation between time and EMS care process.

### Participants

Twenty EMS care providers (twelve males and eight females) gave verbal and written informed consent before the study. Their average age was 44.2 years (SD 8.4), ranging from 28 to 62 y. They had, on average, 10.2 (SD 6.6) years of experience as an EMS nurse, ranging from 1.15 to 25 y. and had, on average, 19.9 (SD 10.0) years of experience as an RN, ranging from 1.4 to 35 y.

### Patient involvement

Neither patients nor the public were not involved in this research’s design, conduct, or reporting since the study focused on the care providers rather than individual patients or patient groups.

### Ethics approval and consent to participate

This study was approved by the Medical Ethical Committee of the University Medical Centre of Groningen (UMCG), the Netherlands (reg.no. M18.224402), as not subject to the Medical Research Involving Human Subjects Act. All methods were carried out according to relevant guidelines and regulations. Twenty EMS care providers (twelve males and eight females) gave verbal and written informed consent before the study.

### Data analysis

The qualitative data analysis of the video observations took place in a deductive manner.

The video footage was analysed using the qualitative data analysis software package Atlas.ti (versions 8/9, Cleverbridge AG, Cologne, Germany) and was independently evaluated by pairs of raters (last year’s nursing students) supervised by the first author (BD). The raters identified professional activities executed by the EMS provider within each case by thoroughly studying the footage. If necessary, the accompanying interviews were used to clarify unclear activities.

After the identification of the activities, they were collated into categories. We used the SPART model as a matrix for the categorisation.

After rating the activities into categories, the degree of agreement of each two raters was examined. In the event of a difference of opinion, a consensus discussion and a final classification of the video interpretation followed. We identified diagnostic, therapeutic, and other activities, measured when and how often they occurred and determined their duration. Watching the video footage also allowed us to recognise when the different logistical phases of a deployment began and ended. We distinguish three time phases within each deployment: the response, on-scene, and transport periods.

The response period is between taking the call and arriving at the scene. The on-scene period is between arrival at the scene and transport start (if applicable). The start of transport marks the beginning of the transport period, which ends with the transfer to another healthcare professional.

To test whether the deployment execution was systematic and coherent, we interpreted the content of the cases by following logical rules, common sense, and clinical experience. Some examples of logical rules we used were: “Data collection must always precede data interpretation”, “The initiation of therapy is always the result of a (clinical) decision”, and “To assess the effect of therapy, it is necessary to reassess the patient’s condition, represented by his appearance, vital signs and subjective feeling”. Once the course of the case became clarified and appeared logical, the clinical reasoning process became clear and could be assessed for medical content.

## Results

### General characteristics of cases

We included 28 clinical cases from 20 participants with a total recorded time of 18h 46m (summarised duration of all deployments). Some participants handled more than one case. To determine whether our sample was representative of typical deployments in the Netherlands, we calculated the average times of the three deployment phases: response period, on-scene period and transport period (Table 1). They were comparable to the Dutch nationwide mean times (all figures 2021) [24], although we could not test this statistically because we did not have access to the Dutch raw data.

**Table 1:** Average times and standard deviation of deployment periods (n = 28) compared to NL averages (2021).

	response period	on-scene period	transport period	deployment time
all deployments	03h 17m 59s	11h 34m 49s	03h 53m 12s	18h 46m 00s
mean	07m 04s	24m 49s	12m 16s	40m 13s
SD	04m 49s	11m 04s	09m 36s	12m 57s
NL nationwide	07m 17s	23m 43s	n/a	45m 45s

The characteristics of the included clinical situations are presented in Table 2. The cases varied sufficiently to represent the normal distribution of EMS-handled clinical cases in the Netherlands [24].

**Table 2:** Included clinical situations.

clinical situations	n
accident	7
chest pain	6
neurology	4
collapse	4
dyspnea	2
haemorrhage	2
unconsiousness	1
violent abuse	1
malaise	1
**total**	**28**

### Quantitative and qualitative analysis

We identified 1683 activities, with a summarised duration of 12h 34m 30s. The diagnostic and therapeutic activities were assigned to one of the ten categories of the SPART model. Activities which could not be assigned to a particular category, e.g., preparation of stretcher before conveyance, were categorized as “other”. Table 3 summarises the quantitative findings for all included deployments together.

**Table 3:** Durations and frequencies of activities within SPART categories.

category	duration	% of total	frequency
Start	00h 52m 48s	7.00%	160
Situation	00h 18m 39s	2.50%	28
Prologue	00h 42m 57s	5.70%	141
Presentation	01h 03m 20s	8.40%	209
Anamnesis	02h 40m 58s	21.30%	322
Assessment	02h 09m 02s	17.10%	293
Reasoning	00h 40m 04s	5.30%	201
Resolution	00h 07m 14s	1.00%	35
Treatment	01h 59m 02s	15.80%	98
Transfer	01h 19m 45s	10.60%	42
Other	00h 40m 41s	5.40%	154
**total**	**12h 34m 30s**	**100.00%**	**1683**

It is noticeable that the diagnostic activities (performed during Situation, Prologue, Presentation, Anamnesis, Assessment, Reasoning and Resolution) took 4.3 times more time than the therapy (Treatment category), namely 08h 35m 02s versus 01h 59m 02s.

**Fig 2:**
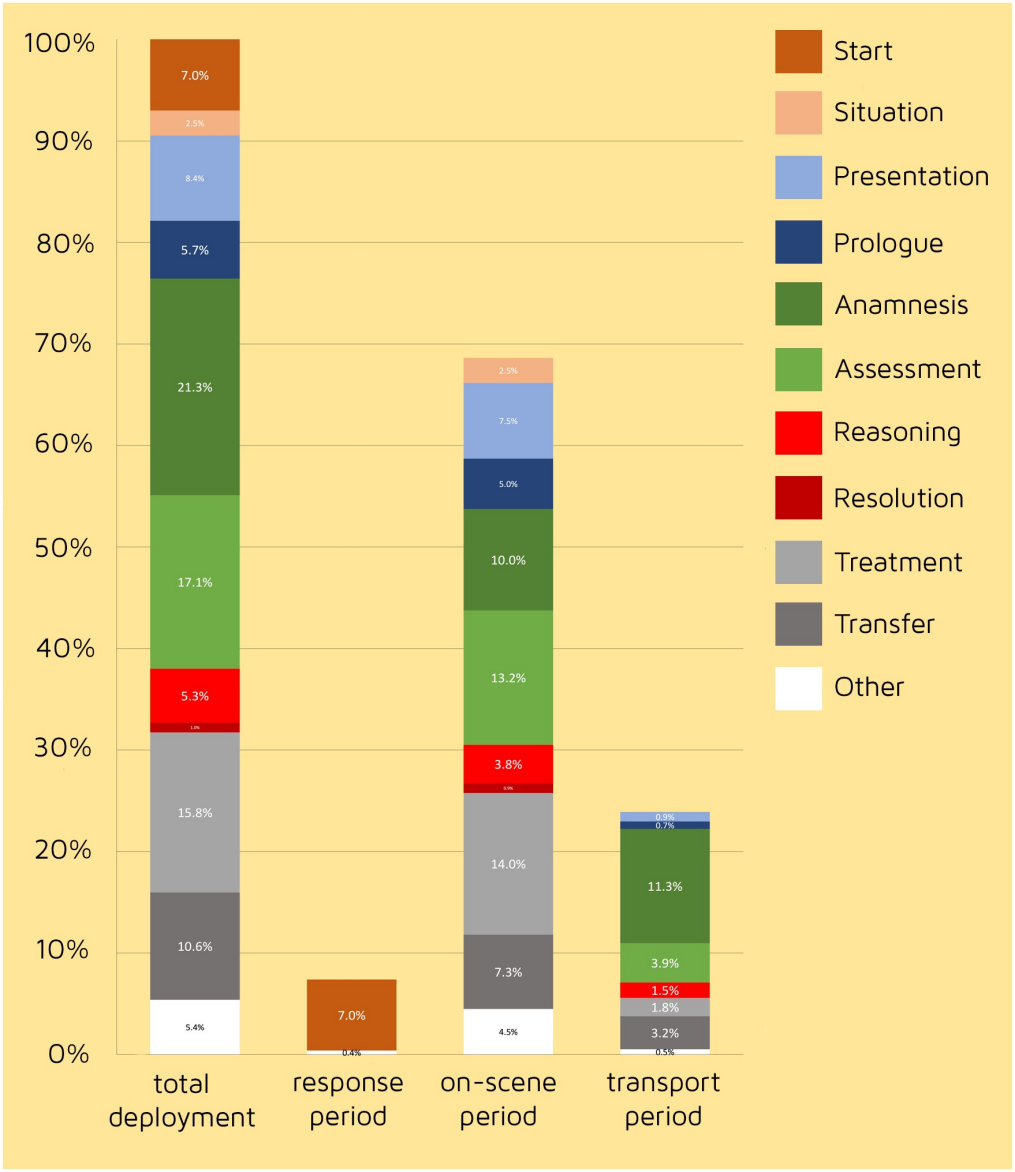
The distribution of the SPART categories over the EMS deployment phases in percentages of real-time duration. (100% = 12h 34m 30s).

Because of the different nature of the three deployment periods, we expected the various SPART categories to be disproportionately distributed across these periods. Fig 2 describes the percentage distribution of the duration of all SPART categories combined (12h 34m 30s = 100%). In addition, it shows the breakdown across the different deployment periods.

Out of these SPART categories, due to its definition, the ‘Start category’ occurs solely during the response period. Since the ‘Situation category’ starts with the first subjective and intuitive interpretation of the scene, it marks, by definition, the beginning of the on-scene period, and therefore, activities within this category are embedded in this specific period. The definition also implies that the activities of the Situation category cannot occur elsewhere during a deployment. Theoretically, activities appointed to the ‘Resolution category’ (the clinical decision) could occur during both the on-scene and transport periods. However, our findings show that the clinical decision was made only once during the on-scene period. Furthermore, it is remarkable that the diagnostic process initiated during the on-scene period continues but decreases in used time during the transport period (respectively 42.9% and 18.3% of total activities time). This is also true for the therapeutic activities (14% and 1.8%).

Also noteworthy is the observation that as deployment time progresses, the emphasis within diagnostic activities almost totally shifts to the general assessment and the general anamnesis, and the share of the P-categories virtually disappears. In the on-scene period, investigating the patient’s primary complaint (Presentation and Prologue) takes 7.5% and 5% of the total activity time, while charting the patient’s general medical status (Anamnesis and Assessment) takes 13.2% and 10%. In the transport period, Presentation and Prologue timeshares decreased to 0.9% and 0.7%, while Anamnesis and Assessment activities still used 11.3% and 3.9%.

We expected diagnostic activities to be performed mainly during the on-scene period. Our data clearly showed that after the formulation of a clinical decision and the start of treatment, the execution of diagnostic activities continues, even after the start of the transport period. An in-depth evaluation of the data set provided insight into this finding. As mentioned, evaluating the primary complaint or symptom (e.g., palpation of the cervical spine, observing that one leg is shortened and asking questions like “What happened?” and “How long have you been short of breath?”) primarily takes place during the on-scene period and dissipates during the transport period. Evaluating the patient’s general medical status, e.g., taking an ECG, asking,”Do you have diabetes?” and “Do you use any medication", continues during the transition of the two final deployment periods. To understand this phenomenon, we further categorized the recognized diagnostic activities in the Transport period.

The activities could be divided into three sub-categories.

1. Questions and physical examination that were forgotten earlier in the deployment.
2. Questions and physical examination that are needed to evaluate the effectiveness of the therapy initiated.
3. Questions to enlighten uncertainties or incompleteness.

In our data, we did not find evidence that the additional information obtained later in a deployment led to a change in the clinical decision. Nor could we show that the "forgotten" questions or the "delayed" examination had adverse medical consequences. Examples are: later still connecting a pulse oximeter (a), “Are you allergic?”(a), remeasuring blood pressure after administrating pain medication (b), and “What is exactly is the nature of your chest pain?”(c).

## Discussion

The primary objective of this study was to investigate the nature of the professional activities within the three EMS deployment periods, focusing on the embedded clinical reasoning process and the relation to the time spent on this.

### The nature and sequence of the activities and structure of the clinical reasoning process

Using the SPART model, we gained quantitative and qualitative insight into the activities carried out during the various deployment periods (response, on-scene and transport) into which an EMS deployment is divided.

The SPART model helped to structure the information and facilitated comprehension of the clinical reasoning process. Generally, the cases in our dataset were reconstructable (i.e. understandable in retrospect), and the sequence of the professional activities was logical and appropriate. A surprise finding was that the diagnostic process occasionally continues after the clinical decision is made and after the transport period has started. Sometimes, information that was inadvertently not investigated or requested at an earlier deployment stage was obtained later. This ‘new’ diagnostic information never led to a change in treatment. Changing the clinical decision was sometimes impossible since its implementation already had consequences. In those cases, the omission may be considered a potentially harmful error. For example, asking, “Are you allergic?” after medication has already been administered is simply too late. Although our data did not show adverse medical consequences, it is conceivable that an omission of this nature could have had consequences for the patient.

### Measuring the duration of professional activities

It was possible to quantify the qualitative interpretations according to the number and duration of activities per operational period. The qualitative data provided insight into the cognitive processes employed during a deployment. Thus, we can evaluate the content of an activity and the time it needs within its context. We wondered whether information on time spent could be helpful to better understand the clinical reasoning process.

This study found that the time used for diagnosis is four times longer than for conducting therapy. It was apparent, that arriving at a working diagnosis and formulating a clinical decision (i.e., the clinical reasoning process) is more challenging than starting a treatment.

Measuring time consumption helped to understand the process of clinical reasoning scientifically better, but examining real-life deployments in this way might be too extensive and time-consuming for daily quality measurement. Therefore, we think detailed time measurement for routine evaluation of the quality of care delivered is unlikely to be helpful.

### The speed-accuracy trade-off

As mentioned earlier, when measuring the quality of care in EMS, much emphasis is placed on the relationship between time spent in operational periods. This seems logical since, for several time-critical conditions, rapid on-scene interventions or fast transport to a facility where immediate therapy can be provided improve treatment outcomes. Thus, patients suffering from time-critical conditions could be better served with a shorter response, on-scene and/or transport periods. Evidence shows that shorter EMS response times improve the outcomes for specific patient categories—for example, non-traumatic cardiac arrest patients [25]. Conversely, several authors demonstrated that prolonged EMS response times were not associated with diminished survival in unselected patient groups [26] and specific groups of trauma patients [27–29]. So, literature addressing the influence of response time on patient outcomes is inconclusive. The effect of prolonged on-scene time is even more unclear, so it is uncertain whether extending it would impair patient outcomes because of its multifactorial nature. Nevertheless, in the Netherlands, regulations still mandate a maximum dispatch-to-hospital time of 45 minutes, putting time stress on all EMS operational periods.

Our results show that too much emphasis on the time factor during medical assistance may be counterproductive. In psychology, the speed-accuracy trade-off [30] describes the inverted relation between the speed of action and accuracy: the faster the action, the higher the probability of errors. There is little insight into the relationship between perceived urgency and the clinical decision-making process in EMS. Since there is much emphasis on the time aspect within EMS care, one might assume that EMS providers sometimes feel compelled to come to a clinical decision and start therapeutic actions even before the diagnostic process has been fully completed. Indeed, in this study, we found evidence for this so-called premature closure phenomenon. Premature closure means a diagnosis is considered final before all evidence is searched for and evaluated. We have not been able to establish whether this is a conscious or unconscious process.

## Conclusion

We noticed that most deployment time is dedicated to diagnostic activities. Our research also demonstrated the vulnerabilities of the clinical reasoning process and revealed the possible influence of the so-called speed-accuracy trade-off.

Our findings point to a possible adverse effect of too much emphasis on time as the key quality indicator in EMS care.

Finally, our research revealed a scientifically interesting perspective on the time consumption of EMS activities, and it also demonstrated the relativity of time and duration of activities. Therefore, we recommend shifting this emphasis on deployment time to a more substantive evaluation of the care provided, focusing on the diagnostic outcome, and do not recommend using time consumption as a key quality indicator for everyday use.

### Limitations

The research was conducted with a restricted demographic scope. This can be seen as a limitation, as international data might have offered new, additional insights. This is a consideration for the future. Although the data are extensive and rich, the number of cases is limited, and we cannot speak with certainty about data saturation.

The dataset was obtained in late 2018 and initially used to develop the SPART model. It has always been the researchers’ intention to further analyze the data in the manner presented in this publication. Since two qualitative analyses were completed sequentially, the time between data collection and publication of the second article took several years. Although we have no reason to believe that the clinical reasoning process within Dutch EMS practice has changed significantly during this time span, the age of the dataset should be taken into account when interpreting the results of this study.

## Supporting information

S1 Data Excel spreadsheet containing the identified professional activities, their position in the timeline and their duration. (XLSX)

S1 Table Detailed description of the SPART categories and examples of activities within these categories. (DOCX)

## Abbreviations

ACH: Ambulancechauffeur” = Ambulance Driver
AVP: “Ambulance Verpleegkundige” = specialized Ambulance Nurse
BMH: “Bachelor Medische Hulpverlening” = Bachelor-degree qualified paramedic
EDR: Electronic Deployment Reports
EMS: Emergency Medical Services
RN: Registered Nurse
SPART: Start, Situation, Prologue, Presentation, Anamnesis, Assessment, Reasoning, Resolution, Treatment and Transfer
