# Supplementary material for: The nature, sequence and duration of professional activities of Emergency Medical Service providers: An observational study to evaluate quality of care using the steps in the EMS care process as described by the SPART model
Source: PLoS One. 2024 Dec 2;19(12):e0311946. doi: 10.1371/journal.pone.0311946 (PMC11611194; doi:10.1371/journal.pone.0311946)
Supplement: S1 Table — (DOCX) [file pone.0311946.s002.docx]

Supplemental material S2

The Spart model clusters activities executed within an EMS deployment into categories

|  | Categories | Activities |
| --- | --- | --- |
| S | Start | -Initiation of the EMS deployment. Emergency call-taking and EMS dispatch. -Interpretation of the information provided by the dispatch centre (first generation of clinical hypotheses). -Pre-Arrival-Preparation (dividing tasks among the crew, anticipating the expected situation on the scene) |
|  | Situation (at arrival) | -First, subjective and intuitive interpretation of the scene. -Ongoing generation of clinical hypotheses: "a wet read diagnosis". -Decision whether acute intervention is necessary. |
| P | Prologue | -Retrospective interpretation of factors leading to and influencing the presenting complaint, injury or health problem -In case of an accident: interpretation of the accident mechanism. |
|  | Presentation (presenting complaint or symptom) | -Indicating the reason for the call for assistance. -Performing focused questioning and targeted physical examination, focused on the primary complaint, injury or health problem. |
| A | Anamnesis | -Medical history taking. -Inventory of medication and allergies. -Identification of treatment restrictions. |
|  | Assessment | -General physical examination. -Assessment of vitals (ECG, BP, HF, RR, SpO_2_). -Neurologic examination, if applicable. -Taking blood samples, if applicable. |
| R | Reasoning, recapitulation | -The process of gathering, ordering, evaluating, and interpreting clinical information to formulate a working diagnosis and consider differential diagnoses. -A clinical time out to overview the gathered information and detect information deficiencies. |
|  | Resolution | -The (clinical) decision on what to do or not to do, based on the working diagnosis and taking into account the patient's wishes, context and options. |
| T | Treatment | -Therapy, if possible, and applicable in the prehospital setting. -Guided by protocols and guidelines. |
|  | Transfer | -Mandatory to conclude the EMS deployment. -Three possible routes: 1. To the patient self. Clinical therapy or conveyance to the hospital or both are not necessary. Shared decision process. Informed consent. With dedicated attention to patients' questions, fears and uncertainties. 2. Handover to other (professional) care provider (i.e., GP, midwife, mental health care provider). 3. Conveyance and handover to a hospital or other care facility. -Evaluation and reflection |
